# Biochemical Composition of Cumin Seeds, and Biorefining Study

**DOI:** 10.3390/biom10071054

**Published:** 2020-07-15

**Authors:** Othmane Merah, Bouchra Sayed-Ahmad, Thierry Talou, Zeinab Saad, Muriel Cerny, Sarah Grivot, Philippe Evon, Akram Hijazi

**Affiliations:** 1 Laboratoire de Chimie Agro-industrielle (LCA), Université de Toulouse, INRA, INPT, 31030 Toulouse, France; bouchra.sayed.ahmad@hotmail.com (B.S.-A.); thierry.talou@ensiacet.fr (T.T.); muriel.cerny@ensiacet.fr (M.C.); sarah.grivot@ensiacet.fr (S.G.); philippe.evon@ensiacet.fr (P.E.); 2 Département Génie Biologique, IUT A, Université Paul Sabatier, 24 rue d’Embaquès, 32000 Auch, France; 3 Research Platform of Environmental Science, Doctoral School of Science and Technology, Lebanese University, Campus Rafic Hariri, BP 5, Hadath-Beirut, P.O. Box 5, Lebanon; zsaad2002@yahoo.com (Z.S.); Akram.Hijazi@ul.edu.lb (A.H.)

**Keywords:** *Cuminum cyminum*, genotypic diversity, vegetable oil, essential oils, by-products, antimicrobial activity, antioxidant activity, sustainability

## Abstract

A new biorefinery approach has been developed in the present study, and applied on cumin (*Cuminum cyminum*) seeds as a potential source of phytochemicals of interest. Cumin is a popular spice used widely for its distinctive aroma. It is a rich reserve of both vegetable and essential oils. The biorefinery approach here focused on the evaluation of the influence of four different geographical origins (i.e., Lebanon, France, Algeria and Syria) on oil yield and quality in cumin seed, and on the valorization of remaining by-products by investigating their nutritional content and biological activity for the first time. Vegetable and essential oils were extracted, and their compositions were determined. Nutritional traits were also assessed. The delipidated and hydrodistillated cakes just as aromatic water were characterized for their fiber, sugar, protein, phenol and flavonoid contents. Antibacterial and antioxidant activities were also determined. Cumin seeds showed high contents in both vegetable and essential oils, proteins and sugars regardless their origin. Moreover, this *Apiaceae* species exhibited high levels of petroselinic fatty acid (an isomer of oleic acid) and sterols. Cakes and aromatic water also presented high levels of proteins, fibers, sugars and phenols. These residues revealed interesting antioxidant and antibacterial activities. These results emphasized the potential use of cumin in a biorefinery concept, with a multi-purpose industrial process. In addition, large differences were observed between the four geographical origins for phytochemical contents and compositions. These findings highlight the perspectives for developing selection programs for nutritional traits and industrial interests. All obtained results validate the health promoting effect of cumin composition as well as its industrial importance along with the residues.

## 1. Introduction

The biorefinery is a fully renewable process that aims to fractionate and capture valuable raw materials from plants. The application of this concept could broaden and diversify products from the agricultural industries, while producing a diverse range of compounds that can be used to support human activities [1].

The *Apiaceae* family, formerly called *Umbelliferae*, includes food plants (carrots, fennels, etc.) and condiments (caraway, coriander, etc.) [2]. Several species of this family are considered to be a rich source of essential and vegetable oils that can be exploited in the pharmaceutical, cosmetic, perfume and food industries [2,3,4,5,6]. The vegetable oil contained in the umbelliferous seeds, which is very rich in petroselinic acid, is localized in the oleosomes. Petroselinic acid (C18:1n12) is a rare monounsaturated fatty acid and isomer of oleic acid which is used as a valuable raw material in the chemical industries [7,8].

In this context, the vegetable oil of several seeds of *Apiaceae* could be a strong competitor thanks to their richness in petroselinic acid [9]. Among these species, cumin (*Cuminum cyminum*) is a promising source of vegetable and essential oils containing high levels of petroselinic acid and other bioactive molecules [10]. Essential oil from cumin was found more efficient than commercial insecticides, and it was proposed in innovative green formulations in crop protection against *E. fetida* and *H. axyridis* [11]. Several studies have suggested to develop nanoparticles based on cumin extracts for effective antleichmaniose [12] and antitumor activities [13]. Indeed, cuminaldehyde presents antinociceptive, antineuropathic and anti-inflammatory effects [14]. However, currently, depending on the industrial field of application, only one of these two fractions is valued, the other constituting a waste. A major scientific question remains the possibility of the sequenced extraction of essential and vegetable oils from cumin seeds. Additionally, the establishment of integrated recovery of cakes appears as a way that can participate in a better use of plant potential while allowing the development of new bioproducts of industrial importance.

**Figure 1:**
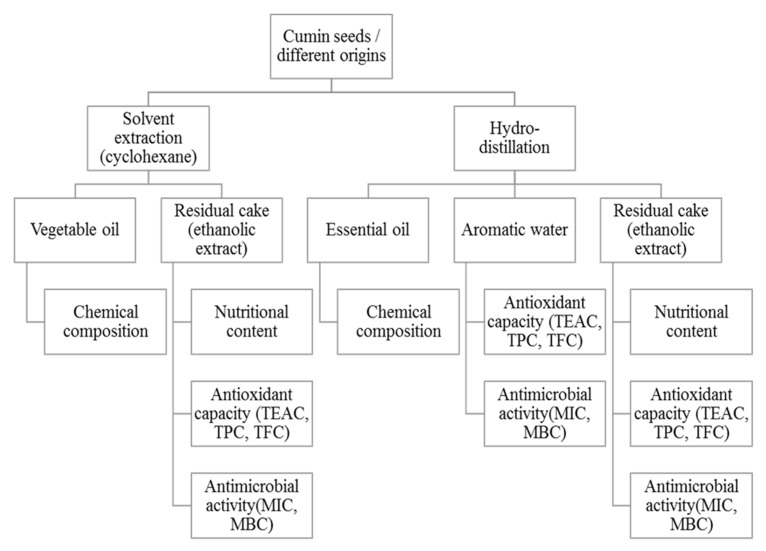
Biorefining process of cumin seeds applied in the present study.

The present work is therefore a contribution in the global valuation of cumin seeds and from a perspective of sustainable development. A new biorefining approach of cumin seed has been established (Figure 1). The plant materials were processed in four parts: the vegetable oil, the essential oil, the aromatic water and the final residue (i.e., the cake). The approach adopted consists first of all of extracting the vegetable and essential oils from cumin seeds from different geographical origins. The residual cakes remaining after extractions and the aromatic water (i.e., the by-products) are later valorized as sources of biosourced molecules (antioxidants or antibacterials). In this way, the molecules inside the cakes can be extracted and used in a sequential way, thus avoiding the wasting of natural resources.

## 2. Materials and Methods

### 2.1. Raw Materials

The *Apiaceae* seeds used in this study were bought on supermarkets, local stores, or in seed companies in four different countries: Lebanon, France, Algeria, and Syria. The solvents and the chemicals, all of analytical grade, were provided by Macherey-Nagel (Germany), Merck (Germany), Prolabo (France), and Sigma Aldrich (USA).

### 2.2. Extraction of Oils and Their Analyses

#### 2.2.1. Extraction of Essential Oil and Its Analysis

Essential oils were extracted using 200 g of cumin seeds in a Clevenger type apparatus for three h, followed by storing at 4 °C. Clevenger extractors are indeed those recommended to extract essential oils at lab scale.

For essential oil composition determination, a HP 5890 series ∏ GC was used, completed by a 5970-mass spectrometer. Two fused-silica capillary columns were used in this study: a HP-5 MS one, and a Carbowax one, both having the same characteristics (i.e., 30 m length, 0.25 mm internal diameter, and 0.25 μm film thickness). Helium was used as carrier gas at flow rate of 0.6 mL/min.

When using the HP-5 MS column, the temperature program was as follows: the initial oven temperature was held at 60 °C for 2 min, after which it increased to 220 °C at a rate of 3 °C/min and maintained at 220 °C for 12 min. When using the Carbowax one, it was as follows: the initial oven temperature was held at 70 °C for 2 min, after which it increased to 220 °C at a rate of 5 °C/min and maintained at 220 °C for 8 min.

The GC-MS parameters were 250 °C for the injector temperature, 280 °C for the ion source temperature, 70 eV for the electron ionization, 30–300 amu and 2.77 scan/s for the mass spectra range, 1/100 for the split ratio, and 1 μL pentane solution for the injection volume.

The individual components in both essential oils and volatile extracts were identified by comparing their retention indices (RI) (determined thanks to an n-alkanes series) with those existing in the literature [15,16,17,18]. The comparison of their mass spectra with those present in the mass spectra library of data process softwares (NBS75 K database, Wiley 7th NIST 98 EPA/NIH Mass Spectral Library, Mass finder 3/Hochmuth, and FFNSC2/Mondello, 2nd Edition, November 2011) completed the identification, just as their comparison with those also existing in published data. The normalization results of peaks appearing in chromatograms led to the relative percentage of each component in the essential oil.

#### 2.2.2. Extraction of Vegetable Oil

A Soxhlet extraction apparatus was used to extract continuously vegetable oil. The extraction was performed by adding cyclohexane as extracting solvent to 25 g grinded powder from cumin seeds during 5 h. Cyclohexane was then removed using a rotary evaporator. Oil was conserved in a dark bottle at 4 °C.

#### 2.2.3. Fatty Acid Profile Determination

The fatty acid profile was determined after dissolution of oil in Methyl-tertbutyl-ether (1 mL added to 20 mg) and conversion into fatty acid methyl esters (FAME) by adding trimethylsulfonium hydroxide (0.2M in methanol) (50 µL). GC/FID was used for analyzing FAME. The capillary column was a CP-Select CB fused silica WCOT one, with following characteristics: 50 m length, 0.25 mm internal diameter, and 0.25 µm film thickness. The temperature program chosen for the analysis was as follows: the initial oven temperature was held at 185 °C for 40 min, after which it increased to 250 °C at a rate of 15 °C/min and maintained at 250 °C for 10.68 min. Both the injector and the detector had the same temperature setting, i.e., 250 °C. The carrier gas used was helium with a flow rate and split ratio of 1.2 mL/min and 1:100, respectively.

#### 2.2.4. Unsaponifiable Compound (i.e., Sterol) Determination inside Vegetable Oils

The saponification of vegetable oil (100 mg test sample mass) was conducted at 75 °C and for 20 min by adding 2 mL of a KOH solution, 10% in ethanol. Cholestanol was used as internal standard. Once the mixture was cooled up to the ambient temperature, distilled water (1 mL) and cyclohexane were added (6 mL). After stirring, 160 μL of the organic phase were taken and completed with 40 µL BSTFA (bis (trimethylsilyl) trifluoroacetamide) and TMCS (trimethylchlorosilane) (99/1, *v*/*v*). The mixtures were analyzed with a PerkinElmer CPG-FID (Waltham, MA, USA). A CP-SIL 8CB capillary column (30 m length, 0.25 mm internal diameter, and 0.52 μm film thickness) and H_2_ as the carrier gas (1 mL/min flow rate) were used. The temperature program chosen for the analysis was as follows: the initial oven temperature was held at 160 °C for 0.5 min, after which it increased firstly to 260 °C at a rate of 20 °C/min and maintained at 260 °C for 5.5 min, secondly to 300 °C at a rate of 2 °C/min and maintained at 300 °C during 10 min, and lastly to 350 °C at a rate of 45 °C/min and maintained at 350 °C during 3 min.

### 2.3. Nutritional Content Determination from By-Products

Moisture and dry matter contents of cumin residues collected after both delipidation and hydrodistillation were determined according to ISO 665:2000 [19]. Their mineral and protein contents were assessed according to ISO 749:1977 [20] and ISO 5983-1:2005 [21], respectively. For protein contents, the conversion factor between total nitrogen and crude protein was chosen equal to 6.25. The anthrone method of Yemm and Willis was used to determine the total soluble sugar content [22]. The Van Soest and Wine method was used to determine neutral detergent fiber (NDF, i.e., hemicelluloses plus cellulose plus lignins), and acid detergent fiber (ADF, i.e., cellulose plus lignins) [23,24].

### 2.4. By-Products and Their Biological and Chemical Analyses

The cakes resulting from both delipidation and hydrodistillation were treated using a Soxhlet extraction apparatus, and ethanol as extracting solvent. The two obtained ethanolic extracts were then freeze-dried, just as the remaining water originating from hydrodistillation (i.e., aromatic water).

#### 2.4.1. Total Phenol Content (TPC) Determination

TPC were determined using the Folin-Ciocalteu [25] method at 765 nm. For each extract, the determinations were made three times, and TPC was deduced from a calibration curve obtained from standard solutions of gallic acid having a 50–500 mg/L concentration range. TPC content was expressed in mg of gallic acid equivalent (GAE) per gram of extract (mg GAE/g extract).

#### 2.4.2. Total Flavonoid Content (TFC) Determination

Aluminum chloride assay through colorimetry [26] was used to determine TFC of cakes from seeds, and residual water. After 15 min incubation, absorbance was determined using a UV-visible spectrophotometer at 510 nm. A blank sample consisting of distilled water was used. A rutin standard curve was used for quantification, and TFC was expressed in mg of rutin equivalents per gram of extract (mg Ru/g extract).

#### 2.4.3. Trolox Equivalent Antioxidant Capacity (TEAC) Determination

A modified method from Brand-Williams et al. [27] was used to determine the radical scavenging activity against stable DPPH radical using a UV-visible spectrophotometer at 515 nm. All measurements were conducted in triplicate. Calibration was made from Trolox methanol solutions having concentrations situated between 100 and 750 μmol/L.

#### 2.4.4. Antibacterial Activity Determination

- Strains of bacteria:

Bacteria used in this study were (i) three Gram-positive strains (CIP 444 *Staphylococcus epidermidis*, ATCC 25923 *Staphylococcus aureus*, and ATCC 29212 *Enterococcus feacalis*), and (ii) two Gram-negative ones (ATCC 35218 *Escherichia coli*, and ATCC 27853 *Pseudomonas aeruginosa*).

- Assays for Minimum Inhibitory Concentrations (MIC), and Minimum Bactericidal Concentrations (MBC):

A microtiter broth dilution method [28] was used to determine both MIC and MBC concentrations. A 96-well plate (200 μL Per Well) (Corning^®^ Costar^®^ 3598; Corning, NW 14831, USA) was used for the preparation in MHB (Mueller Hinton Broth) of serial two-fold dilutions of the different extracts. A positive growth control was also considered in the form of wells with no extract added. For each strain, a diluted bacterial suspension was obtained and adjusted to a 5 × 10^5^ Colony-Forming Units (CFU)/mL final concentration. A negative growth control was also considered in the form of wells with no bacterial inoculum. Duration and temperature of the incubation of plates were 24 h and 37 °C, respectively. MBC was determined by counting the number of colonies after overnight incubation at 37 °C. The MIC and MBC concentrations were determined for all strains. At least three independent determinations were made for each of them.

### 2.5. Statistical Analyses

The experiments in this study were all conducted in triplicate. Results were defined as mean values ± standard deviations. The different individual means were compared between them using one-way ANOVA and Tukey tests. The chosen probability level was 5%. MS Excel 2010 software was used for determination of the R^2^ linear correlation coefficients.

## 3. Results

### 3.1. Yield and Chemical Composition of Essential Oils of Cumin Seeds

Yield and chemical compositions of the essential oils of cumin seeds from Lebanon, France, Algeria and Syria are presented in Table 1. Two samples were tested for cumin seeds of each geographic origin. Standard deviations for all identified volatile compounds in each batch were considered as negligible, and this is the reason they are not mentioned in Table 1.

The yield of essential oil varied between 1.6% and 2.9%, depending on the origin of the seeds. Higher value was observed for the Syrian origin. In contrast, Algerian cumin showed the lowest yield.

Twenty-five components representing about 97% of the essential oil were identified in the four samples (Table 1). The chemical classes of the oils showed the prevalence of oxygenated monoterpenes (66.5% for the French oil, and up to 66.9% for the Algerian one), represented mainly by cuminaldehyde (43.9% and 49.5%, respectively) and 1,4-p-menthadien-7-al (17.2% and 17.1%, respectively). Monoterpene hydrocarbons constituted the second largest class, β-pinene, p-cymene and γ-terpinene being the major constituents of this class. The fresh and spicy aroma of cumin essential oil was related to γ-terpinene, cuminaldehyde and menthane derivatives.

### 3.2. Vegetable Oil Content, Fatty Acid and Phytosterol Compositions in Cumin Seeds

The values of vegetable oil yields and fatty acid composition of cumin seeds from different origins are shown in Table 2. The yields varied more than two times between extreme origins. The French population revealed a significantly higher yield than the three other ones.

MUFA, monounsaturated fatty acids; PUFA, polyunsaturated fatty acids; SFA, saturated fatty acids.

**Figure 2:**
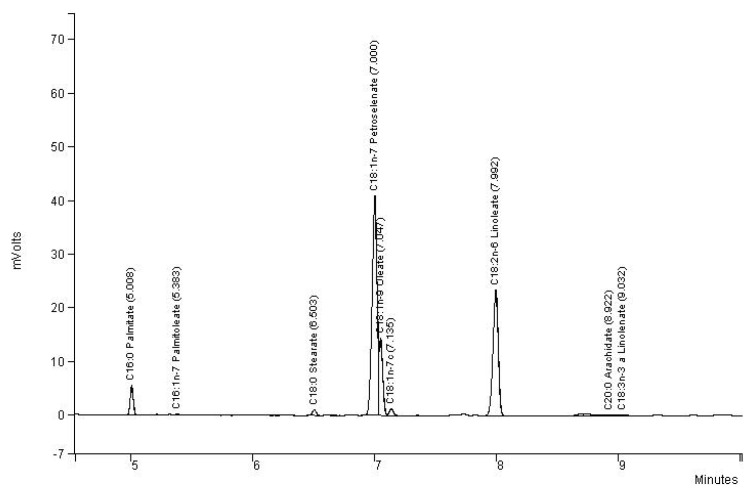
Chromatogram of fatty acid composition of Lebanese population of cumin.

Nine fatty acids were identified (Figure 2). In particular, despite very close retention times, two different peaks appeared for petroselinic and oleic acids. For its part, linoleic acid appeared at a much higher retention time. Petroselinic acid (C18: 1n-12) was the most important fatty acid for the four varieties. The seeds of French and Algerian origins showed significantly higher levels (51.5% and 51.6%, respectively) of this specific fatty acid than the two other populations. Linoleic acid was the second most important component identified whatever the origin, followed by oleic acid and then palmitic one (Table 2).

Sterol composition of cumin vegetable oils from different origins are shown in Table 3. Four compounds were identified in those various oils. As expected, β-sitosterol was the major component found in all samples (Table 3). Syrian cumin showed 50% more sterols than Lebanese one.

### 3.3. Nutritional Content of Cumin Seeds

Nutritional composition of cumin seeds is shown in Table 4. A significant difference in the total mineral content was observed between the different origins of cumin. Lebanese seeds presented two times higher mineral content than the French ones.

The protein content in the various seeds varied significantly, i.e., from 20.9% in the Algerian variety to 24.7% in the Lebanese one. The variation of soluble sugars between the different origins followed the same trend than the two former traits. The Syrian variety showed the highest value while that of France contained the lowest one.

### 3.4. Effect of Hydrodistillation and Delipidation on Cumin Cakes

The nutrient contents of residual cakes resulting from the extraction of essential oils or vegetable oils are presented in Table 5. The results showed the richness of these cakes from different varieties in minerals, proteins and soluble sugars, particularly in the case of delipidated cakes.

The ADF and NDF fiber contents in the residual cakes of cumin seeds are also presented in Table 5. These contents varied considerably between the different cake samples. In particular, those from seeds originating from France were the least rich in NDF and the richest in ADF. This suggests that cakes from seeds of French origin were the poorest in hemicelluloses but also the richest in lignocellulose, especially the delipidated one. Delipidated cakes showed higher values than the hydrodistillated ones. However, all cakes of different varieties can be considered as naturally rich in fibers.

### 3.5. Biological Activities of Cumin By-Products

#### 3.5.1. Total Contents of Phenols (TPC), Flavonoids (TFC) and Antioxidant Activity

Since phenolic compounds are major factors in the antioxidant activity of plants, the contents of TPC and TFC in cumin cakes and aromatic water (i.e., the remaining water after hydrodistillation) were determined in this study. The results are shown in Table 6. All samples presented high levels of TPC and TFC regardless the geographic origin. However, studied samples differed in the phenol and flavonoid contents. Indeed, the by-products from both French and Syrian cumin showed the highest values. The aromatic waters were the richest in phenols and flavonoids, followed by the delipidated cakes and then the hydrodistillated ones (Table 6).

The results in Table 6 show the potent antioxidant capacity of all residues, especially those of cumin of French and Syrian origins. Moreover, among the different types of residues studied, aromatic waters have the highest antioxidant activity. Here, the results indicate that different cumin residues have good antioxidant potential, thus appearing as rich and novel sources of natural antioxidants.

A positive correlation was found between TPC and TFC values (R^2^ = 0.85 **, *p* ≤ 0.01), and between TFC and TEAC values (R^2^ = 0.94 **, *p* ≤ 0.01).

#### 3.5.2. Minimum Inhibitory and Bactericidal Concentrations (MIC and MBC)

The MIC and MBC concentrations of Gram-positive and Gram-negative bacteria of the residual cakes originating from the extraction of vegetable and essential oils from French cumin were examined, and the results are summarized in Table 7. This table also contains the results obtained from the aromatic water. Delipidated cake showed variation in its antimicrobial activity. However, *S. epidermidis* and *E. coli* were the most susceptible. Hydrodistillated cake and aromatic water showed no inhibitory or bactericidal effect against the different strains. The applied concentrations were much lower than the MIC and MBC concentrations of the bacteria present.

## 4. Discussion

### 4.1. Yield and Chemical Composition of Essential Oils of Cumin Seeds

Data on lipids and essential oils from Lebanon, France, Algeria and Syria have not yet been undertaken. Thus, in the present study, the analysis of these varieties was conducted in order to enhance their valuation as new sources of vegetable and essential oils.

Differences in essential oil yield were observed between the seeds of the four origins (Table 1). The examination of cumin seeds from different geographical areas has been the subject of several previous studies. Comparative yields were obtained for Indian (1.2–1.9%) [29,30] and Egyptian (2.5%) cumin seeds [31]. Lower yields were found for Iranian (1.5%) and Tunisian (1.2%) cumin [32]. In contrast, cumin seeds from China gave a higher yield (3.8%) [33]. Cumin growing in agro-ecological sub regions (India) exhibited essential oil yield ranging from 2.1% to 4.5% according to the climatic conditions and region [34]. Moreover, variations in the yield of cumin essential oil during maturation were observed, and ranged from 1.9% to 2.3% for ripe and immature fruits, respectively [35]. Various yields (from 0.6% to 1.4%) were also obtained by different extraction methods [36]. These differences in yield of essential oil can be attributed to genetic factors, stage of seed maturity, and environmental factors [37,38].

Twenty-five components were identified in our study (Table 1). In comparison, in Egyptian cumin, twenty-one constituents, representing 90.2% and 95.6% of grass and seed oils, respectively, were identified [35]. Forty components were identified in Tunisian and Indian cumin essential oils, of which thirty-four were present in both oils [27]. Our results agree with those already reported, where cuminaldehyde was also identified as the main component in the various cumin oils analyzed [33,35,39]. Similarly, Derakhshan et al. [40] found that the main constituents of the essential oil of *C. cyminum* were cuminaldehyde and methane derivatives. In contrast, Spanish cumin essential oil was dominated by γ-terpinene [41], whereas α-pinene and limonene were the main components of Iranian cumin [42]. In fact, the composition of the essential oil of cumin depends on many factors, such as the harvest period, the extraction method, the variety, the geographical origin and the storage conditions [43]. It is important to note that the main components of the different essential oils reported in the literature are also identified in our study (Table 1).

### 4.2. Vegetable Oil Content, Fatty Acid and Phytosterol Compositions in Cumin Seeds

Vegetable oil yield ranged between 13.4% (Algeria) and 29.1% (France) (Table 2). Results obtained for Algerian seeds are quite similar to those obtained for cumin from Pakistan (18.7%) [44], from India (14.5%) [45], and from for Tunisia (15.4%) and India (17.7%) [46]. However, Mallet et al. reported high vegetable oil yield in French seeds (18.4%) [47]. Here, French seeds contained more than 29% oil. This difference may be explained by genotypic or environmental factor [48]. Rebey et al. found a significant increase in total lipid content in cumin seeds during maturation (from 8.2% to 16.9%) [49]. The vegetable oil yield of cumin seeds can vary from 11.5% to 15.2%, depending on the grinding method used [50]. In parallel, the effect of salinity on cumin production resulted in a decrease of oil yield for Tunisian cumin [31]. This diminution is probably due to the decrease of activity of enzymes involved in lipid biosynthesis [49].

The main fatty acids present in cumin seeds were petroselinic and linoleic acids regardless the origin (Table 2). Petroselinic acid is known to be a general characteristic of the seed oils of *Apiaceae* species, representing up to 72.6% in the case of a coriander vegetable oil [3,8,51]. In this study, the level of petroselinic acid in the studied cumin vegetable oils are comparable to those found in cumin from Tunisia (55.9%) [38,46], and from Pakistan (51.3%) [44]. In contrast, Bettaieb et al. [46] and Rebey et al. [49] found lower rates in Indian varieties (41.4%) and immature cumin seeds (10.6%). Hemavaty and Prabhaker found a much higher content in Indian cumin (83.4%) [45] than in all other studies. In contrast, salinity reduces petroselinic acid content in Tunisian genotype [38]. Salt effect was reported to inhibit the biosynthesis of lipids. This inhibition is mostly due to the decrease of the activities of desaturase enzymes [41].

Petroselinic and linoleic acids and the other monounsaturated fatty acids (MUFA) accounted for more than 60% of total fatty acids whatever the geographic origin (Table 2). Actually, MUFA can reduce cholesterol (LDL), while they could eventually increase the content of high-density lipoprotein (HDL). Petroselinic acid is of potential industrial importance. It can be cleaved by oxidation to produce a mixture of lauric acid, a compound very useful in the production of detergents, and adipic acid, a dicarboxylic acid that can be used in the synthesis of the nylon polymer [52]. Coriander seed oil, due to its richness in petroselinic acid, obtained in 2013 authorization as a Novel Food Ingredient (NFI) from the European Food Safety Authority (EFSA) [53]. In addition, oleic acid can promote insulin resistance. It is identified as a suitable biomarker for studying the relationship between the metabolic profile and the risk of breast cancer [54]. Polyunsaturated fatty acids, represented mainly by linoleic acid, made up more than 30% of the total fatty acids (Table 2) in all samples. Linoleic acid is one of the essential fatty acids that are considered as additives in functional foods and nutraceuticals. Numerous studies have documented the significant roles of essential fatty acids in many biochemical pathways that result in a cardioprotective effect. Their considerable effect on reducing the risk of serious diseases such as cancer, osteoporosis, diabetes and others has also been reported [55].

Four sterols were identified in cumin seeds of all origins, with the main one which was β-sitosterol, the latter representing 43% to 47% of total sterols depending on the origin (Table 3). These results are in agreement with those obtained by Zlatanov et al., where 45.6% of β-sitosterol and 39.7% of stigmasterol were found in cumin vegetable oil [56]. In addition, Ramadan et al. showed that β-sitosterol and stigmasterol represented 25.8% and 25.1%, respectively, of the total sterol content in cold-extracted cumin oil [57]. Campesterol and Δ^5^-avenasterol were also detected in the four samples but at much lower levels, which did not correspond with the results of Ramadan et al. who found 24.3% of Δ5-avenasterol in cumin oil [57]. However, it has been reported that the concentration of sterol components may be affected by environmental factors and harvest dates [48,58,59]. Phytosterols, in general, are interesting due to their antioxidant activity and their positive impact on human health. Recently, there is a growing trend towards the use of sterols as additives for functional foods production [58].

### 4.3. Nutritional Content of Cumin Seeds

The evaluation of the contents in minerals, proteins and soluble sugars of cumin is crucial in order to identify the varieties with the best nutritional contents. High contents were observed for nutritional traits in cumin seeds of all origins. Moreover, origins differed for these traits (Table 4).

The high mineral content found in Lebanese cumin is close to that of Iranian one (9.5%) [57,60,61]. In addition, the levels found in Algerian and Syrian cumins are comparable to those found in Egyptian cumin (7.7%) [61] and in Indian cumin (7.3 to 8.0%) [60,62]. These values are below the maximum limit for cumin seeds according to the International Standards Organization (ISO: 9301/2003): 12%.

Protein content ranged from 20.9% to 24.7%, depending on origin (Table 4). These values are in agreement with the study of Khan et al., who found 22.4% of proteins in Indian cumin [40]. However, these levels were higher than those obtained in five previous studies who reported a range of 15.7% to 19.8% [24,61,63,64,65].

Soluble sugars presented a large variation between the four tested origins (Table 4). These values were higher than those previously reported in Indian cumin, just as in a collection of Iranian populations [60,62,66]. These differences may be explained by environmental and/or genetic effects [66]. It has been reported that drought, salinity, low temperatures and floods may increase the concentrations of soluble sugars, while high light irradiance, heavy metals, nutrient scarcity and ozone can decrease these concentrations in seeds. Nevertheless, changes in sugar content do not follow a static pattern and may vary with genotypes and stresses [67].

The observed results were specific to this study and comparisons could be indicative. Nevertheless, it is important to mention that the chemical composition of cumin seeds varies considerably depending on the variety, the cultivation practices, the planting season, the stage of development, and the climatic conditions [48,63].

### 4.4. Effect of Hydrodistillation and Delipidation on Cumin Cakes

Chemical characterization of cakes resulting from delipidation and hydrodistillation was performed. Results showed that delipidated cake was richer in nutrient than cake obtained after hydrodistillation. Moreover, origin of cumin seeds affected the biochemical composition (Table 5).

Very little information is available in the literature on the residual cakes from cumin seeds. Sowbhagya et al. reported that partially delipidated cumin residues contain 20% proteins and 5% minerals [68], while the hydrodistillation residues contain 19% proteins and 9% minerals [69]. The soluble and insoluble dietary fibers constitute the polysaccharides storage of plant cell walls, which cannot be hydrolyzed by human digestive enzymes. The consumption of dietary fibers has attracted much attention, due to their role in preventing cardiovascular diseases, diabetes, colon cancer and obesity. A diet that provides an adequate amount of fibers is usually less energy dense and bulkier, and therefore it can bring a sense of satiety sooner. In addition, the fibers help to eliminate waste and improve the health of the colon. They help to feel full, favoring weight control and regulation of blood sugar levels [70]. The results in this study related to soluble and insoluble dietary fibers (Table 5) are comparable with those obtained by Khan et al., where caraway was the richest in fibers among several spices studied (55.2% NDF, and 24.1% ADF) [60]. In addition, Sowbhagya et al. showed that residual cakes from the extraction of volatile compounds are very rich in dietary fibers [68].

Thus, cumin cakes may have potential uses in various food formulations, showing improved digestibility and good nutritional composition. Different sources of cakes have been used for protein fortification in bread and therefore increased the nutritional and technological attributes [4,5]. Cumin, fennel and caraway cakes supply resulted in higher nutritional values, antioxidant activities and acceptability by a panel of consumers [4,71,72].

Moreover, with respect to the biorefinery concept, one other original application of cumin cakes could be the production of binderless fiberboards through hot pressing, with proteins acting as natural binder and fibers as mechanical reinforcement. These boards could possibly be usable as furniture or building materials, with respect to the indoor air quality. In particular, delipidated cakes would be of real interest for such non-food application, due to their higher protein and fiber contents. Such a valuation has already been highlighted with success using cakes originating from two other *Apiaceae* seeds, e.g., fennel [73] and coriander [9,74]. In addition, in the specific case of the coriander-based boards, an emission of terpenoid compounds was evidenced [75], providing these boards with a significant added value, owing to their characteristic fragrance and neurological, antioxidant and antimicrobial activities. A more recent study also evidenced that no formaldehyde was emitted from these boards, making them much more environmentally friendly materials in comparison with commercial wood-based panels (e.g., OSB, MDF, plywood, and chipboard) [76].

### 4.5. Biological Activities of Cumin By-Products

#### 4.5.1. Total Contents of Phenols (TPC), Flavonoids (TFC) and Antioxidant Activity

Studied samples showed high levels of TPC and TFC. Moreover, a difference was observed between origins for phenol and flavonoid contents (Table 6). French and Syrian by-products of cumin exhibited the highest values for these traits. The richest samples were the aromatic waters.

The important values of TPC and TFC in delipidated cakes, especially the one of French origin, can be attributed to the fact that cyclohexane does not lead to efficient extraction of phenolic compounds, due to their high polarity. The main phenolic compounds remain intact in the residue after defatting [31,77]. The results in this study were in accordance with those of already published reports. Chen et al. found that the hydrodistillation residues of Chinese cumin contained 19 mg GAE/g [78]. Muthamma Milan et al. showed that hot water extracts from cumin were the richest in TPC. These differences could be attributed to genetic factors, ecophysiological conditions, or differences in extraction methods [69]. Bettaieb Rebey et al. found that the TPC and TFC values of Indian and Tunisian cumin seeds varied significantly, depending on the geographical origin and on the extraction solvent used [79]. Catechin, quercitin and rutin were found to be the most important flavonoids in Algerian cumin [77].

Potent antioxidant activity was reported for all samples, the most important was that of aromatic water. French and Syrian residues showed the highest antioxidant activities whatever the residual samples (Table 6).

The antioxidant activity is one of the most important functional activities of cumin that has been noticed in several studies. Many studies have focused on the analysis of the antioxidant activity of extracts and cumin essential oils [77,80,81,82,83]. Nevertheless, the residual cakes did not receive much importance. Chen et al. found that hydrodistillation residues have the highest antioxidant activity among the various extracts analyzed [78]. The TEAC value analyzed by Shan et al. on the methanolic extract of Turkish cumin was 66.1 TE μmol/g [84]. Damasius et al. evaluated the antioxidant properties of aqueous and ethanolic cumin extracts, and they found that the aqueous extract had a greater DPPH activity than the ethanolic one [85], which is in line with the results in the present study. In addition, results obtained by Bettaieb-Rebey et al. indicate that aqueous solvents are the most suitable for the extraction of DPPH free radical scavengers from cumin seeds of several origins [78]. Similar relationships have been obtained in previous studies [84,85,86], which proves that phenolic compounds in cumin cakes significantly contribute to their antioxidant capacity.

#### 4.5.2. Minimum Inhibitory and Bactericidal Concentrations (MIC and MBC)

Antibacterial activities of residual cakes and aromatic water from cumin seeds of French origin were investigated, and the results showed an effective inhibition of pathogenic bacteria for methanolic extract of delipidated cake (Table 7). The most affected bacteria were *Escherichia coli* and *Staphylococcus epidermidis*. In addition, no activity was observed for hydrodistillated cake (Table 7).

Interest in plants with antimicrobial properties has been revived because of drug resistance associated with the use of antibiotics. Nowadays, several crude extracts of plants have been studied for their potential antimicrobial activity or for the identification of new antibacterial agents. The antibacterial activity of cumin seeds has been demonstrated against a broad spectrum of Gram-positive and Gram-negative bacteria [87]. Methanolic extract of cumin seeds was found to present an effective biocidal effect on *Staphylococcus aureus* [77,88]. No information was found regarding the antibacterial activity of cumin residues. The results in the present study are in agreement with previous studies which showed that ethanol was a better solvent for the extraction of antimicrobial active substances than water [89]. The absence of the activity of the hydrodistillated cake can be attributed to the dissolution of the active molecules in the essential oil. Several researchers have already reported that essential oils are the main contributors to the antibacterial activity of cumin seeds, thanks to their wealth of bioactive molecules, including cuminaldehyde [29,90].

## 5. Conclusions

Oil yield and composition of cumin seeds were definitely geographical origin-dependent. Our overall results indicate a good quality of cumin oil that could be used for functional food applications as well as for cosmetic, scented and pharmaceutical applications. Moreover, nutrient contents were significantly affected by the geographical origin of the seeds. The analysis of the biological activity of the cakes shows that the process of delipidation does not affect their antioxidant and antibacterial capacities. However, the bacteria used in the present study were less sensitive to hydrodistillated cake extracts, since the antibacterial activity of seeds is generally due to essential oils, which play a key role in the defense mechanism of seeds against pathogenic bacteria. A comparison of the results leads to the conclusion that the constituents of cumin seed and cake can serve as a nutrient source as well as drugs useful in the chemotherapy of certain infections caused by bacteria, and also as an antioxidant agent. This work suggests potential applications for practical uses of cumin seed extracts and their by-products that could then be implanted as a low-cost renewable source in various industrial areas.

## Figures and Tables

**Table 1:** Yield and chemical composition of essential oil for cumin seeds of four different geographic origins.

Nb.	Volatile Compounds	RI	RT	Lebanon	France	Algeria	Syria
	Monoterpene Hydrocarbons	(%)					
1	α-Thuyene	927	6.65	0.30	0.19	0.13	0.20
2	α-Pinene	933	6.87	0.57	0.71	0.27	0.40
3	Sabinene	973	8.16	0.51	0.53	0.26	0.38
4	β-Pinene	977	8.29	11.49	12.61	10.56	8.21
5	Myrcene	991	8.73	0.62	0.40	0.33	0.51
6	*p*-Cymene	1025	10.01	13.77	10.04	10.30	7.77
7	D-Limonene	1028	10.16	0.53	0.39	0.31	0.49
9	γ-Terpinene	1058	11.37	10.60	6.44	8.67	13.14
12	E-Myroxide	1165	15.93	nf	0.05	nf	nf
14	Terpinen-4-ol	1177	16.42	1.11	0.20	1.33	1.35
15	Terpineol	1190	17.01	nf	0.09	nf	nf
20	Phellandral	1274	20.70	0.15	0.05	0.04	0.29
	Oxygenated monoterpenes	(%)					
8	Eucalyptol	1031	10.26	0.30	0.16	0.19	0.17
10	L-Fenchone	1088	12.59	0.07	0.03	nf	0.04
11	Trans-pinocarveol	1138	14.73	0.08	0.10	0.06	nf
13	Menthol	1167	15.99	nf	0.18	nf	nf
17	Cumenol	1230	18.77	nf	0.04	nf	0.07
18	Cuminaldehyde	1243	19.32	43.89	43.93	49.53	36.19
19	Carvone	1244	19.39	nf	0.36	nf	0.62
21	1,3-p-Menthadien-7-al	1284	21.12	4.40	4.41	nf	8.88
23	1,4-p-Menthadien-7-al	1292	21.40	9.82	17.22	17.07	17.47
24	p-Mentha-1,4-dien-7-ol	1328	23.05	nf	0.08	0.10	nf
	Phenylpropanoids	(%)					
16	Estragol	1198	17.35	nf	0.10	0.03	nf
22	*cis*-Anethole	1285	21.21	0.24	0.55	0.21	0.71
25	Eugenol	1358	24.32	nf	0.11	nf	nf
Monoterpene hydrocarbons				39.65	31.07	32.20	32.68
Oxygenated hydrocarbons				58.56	66.51	66.95	63.44
Phenylpropanoids				0.24	0.76	0.24	0.71
Essential oil yield (%DM)				1.9 ^b^ ± 0.0	1.7 ^bc^ ± 0.0	1.6 ^c^ ± 0.0	2.9 ^a^ ± 0.1

^a–c^ Mean values (of two replicates) followed by different letters in the essential oil yield line are significantly different; nf, not found; RI, [15,16,17,18]; RT, retention time.

**Table 2:** Fatty acid composition (%) determined by GC/FID of cumin seeds of four different geographic origins.

Fatty Acid	Lebanon	France	Algeria	Syria
Palmitic acid (C16:0)	4.2 ^a,b^ ± 0.0	3.9 ^b^ ± 0.2	3.9 ^b^ ± 0.1	4.3 ^a^ ± 0.0
Palmitoleic acid (C16:1n7)	0.3 ^a^ ± 0.0	0.3 ^a^ ± 0.0	0.2 ^a^ ± 0.0	0.3 ^a^ ± 0.0
Stearic acid (C18:0)	0.3 ^b^ ± 0.0	nf	0.9 ^a^ ± 0.0	1.0 ^a^ ± 0.1
Petroselinic acid (C18:1n12)	49.2 ^a,b^ ± 0.4	51.5 ^a^ ± 0.9	51.6 ^a^ ± 0.5	47.4 ^b^ ± 0.9
Oleic acid (C18:1n9)	11.9 ^b^ ± 0.1	11.2 ^b^ ± 0.5	11.3 ^a,b^ ± 0.0	12.2 ^a^ ± 0.9
*cis*-Vaccenic acid (C18:1n7)	1.3 ^a^ ± 0.0	1.5 ^a^ ± 0.2	1.3 ^a^ ± 0.0	1.2 ^a^ ± 0.0
Linoleic acid (C18:2n6)	32.2 ^a,b^ ± 0.1	31.4 ^a,b^ ± 0.7	30.5 ^b^ ± 0.5	32.9 ^a^ ± 0.1
Arachidic acid (C20:0)	0.1 ^a^ ± 0.0	0.1 ^a^ ± 0.0	nf	0.1 ^a^ ± 0.0
Linolenic acid (C18:3n3)	0.5 ^a^ ± 0.0	0.2 ^b^ ± 0.1	0.3 ^b^ ± 0.0	0.6 ^a^ ± 0.0
MUFA PUFA SFA	62.7 32.7 4.6	64.5 31.6 4.0	64.4 30.8 4.8	61.1 33.5 5.4
Vegetable oil yield (% DM)	23.1 ^b^ ± 0.2	29.1 ^a^ ± 0.8	13.4 ^c^ ± 0.2	14.6 ^c^ ± 0.5

^a–c^ Mean values (of three replicates) followed by different letters in the same line are significantly different; nf, not found.

**Table 3:** Phytosterol composition (mg/100 g oil) measured in cumin seeds from four different geographic origins.

Sterol	Lebanon	France	Algeria	Syria
Campesterol	26.3 ^b^ ± 1.4	37.7 ^a^ ± 1.0	39.5 ^a^ ± 1.9	41.6 ^a^ ± 2.9
Stigmasterol	90.9 ^b^ ± 4.5	142.8 ^a^ ± 4.9	139.7 ^a^ ± 8.8	141.9 ^a^ ± 3.3
β-sitosterol	110.3 ^b^ ± 2.6	160.2 ^a^ ± 2.7	153.4 ^a^ ± 12.8	154.8 ^a^ ± 2.1
Δ^5^-avenasterol	9.5 ^b^ ± 0.6	14.9 ^a^ ± 0.3	17.0 ^a^ ± 1.4	18.2 ^a^ ± 2.7
Total	237.0	355.7	349.5	356.6
β-sitosterol/campesterol	4.2	4.2	3.9	3.7

^a,b^ Mean values (of three replicates) followed by different letters in the same line are significantly different.

**Table 4:** Nutrient content, expressed as a mass percentage of the dry matter (% DM), for cumin seeds from four different geographic origins.

Nutritional trait	Lebanon	France	Algeria	Syria
Minerals (% DM)	10.5 ^a^ ± 0.1	5.4 ^d^ ± 0.1	8.0 ^c^ ± 0.1	8.7 ^b^ ± 0.1
Proteins (% DM)	24.7 ^a^ ± 0.3	22.6 ^b^ ± 0.4	20.9 ^c^ ± 0.4	24.0 ^a,b^ ± 0.2
Soluble sugars (% DM)	10.8 ^b^ ± 0.3	8.9 ^c^ ± 0.2	11.2 ^b^ ± 0.1	13.2 ^a^ ± 0.2

^a–d^ Mean values (of three replicates) followed by different letters in the same line are significantly different.

**Table 5:** Minerals, proteins, and soluble sugars contents, expressed in mass percentage of the dry matter (% DM), as well as NDF and ADF contents of hydrodistillated and delipidated cakes from cumin seeds of four different geographic origins.

Source	Trait	Origin			
		Lebanon	France	Algeria	Syria
Hydrodistillated cake (%)	Minerals	9.3 ^a^ ± 0.4	5.1 ^c^ ± 0.1	7.7 ^b^ ± 0.2	6.6 ^b^ ± 0.2
	Proteins	21.4 ^a^ ± 0.3	20.1 ^b^ ± 0.0	16.5 ^c^ ± 0.4	20.3 ^a,b^ ± 0.1
	Soluble sugars	2.2 ^a^ ± 0.1	2.1 ^a,b^ ± 0.0	1.8 ^c^ ± 0.1	1.9 ^b,c^ ± 0.0
	NDF	57.0 ^a^ ± 0.3	50.4 ^b^ ± 0.6	56.9 ^a^ ± 1.0	58.1 ^a^ ± 0.8
	ADF	22.9 ^c^ ± 0.5	35.6 ^a^ ± 0.6	26.7 ^b^ ± 1.2	28.2 ^b^ ± 0.6
Delipidated cake (%)	Minerals	11.0 ^a^ ± 0.0	5.9 ^d^ ± 0.1	8.5 ^c^ ± 0.0	9.8 ^b^ ± 0.2
	Proteins	30.1 ^a^ ± 0.3	26.6 ^b^ ± 0.5	22.9 ^d^ ± 0.1	25.0 ^c^ ± 0.2
	Soluble sugars	12.5 ^b^ ± 0.5	9.5 ^c^ ± 0.2	12.5 ^b^ ± 0.5	14.6 ^a^ ± 0.5
	NDF	59.9 ^a^ ± 0.5	53.2 ^b^ ± 0.8	58.0 ^a^ ± 0.3	59.3 ^a^ ± 0.4
	ADF	25.6 ^b^ ± 1.4	42.1 ^a^ ± 0.8	28.7 ^b^ ± 0.9	28.7 ^b^ ± 0.3

^a–d^ Mean values (of three replicates) followed by different letters in the same line are significantly different.

**Table 6:** Total phenolic content (TPC), total flavonoid content (TFC), and Trolox equivalent antioxidant capacity (TEAC) of delipidated and hydrodistillated cumin cakes, and aromatic water of four different geographic origins.

Trait	Origin	Delipidated Cake	Hydrodistillated Cake	Aromatic Water
TPC (mg GAE/g extract)	Lebanon	31.1 ^b^ ± 0.1	19.8 ^c^ ± 0.1	67.5 ^a^ ± 0.4
	France	69.4 ^b^ ± 0.5	20.2 ^c^ ± 0.1	97.3 ^a^ ± 0.1
	Algeria	24.1 ^b^ ± 0.1	10.3 ^c^ ± 0.8	36.0 ^a^ ± 0.0
	Syria	57.2 ^c^ ± 0.0	59.6 ^b^ ± 0.4	67.5 ^a^ ± 0.3
TFC (mg Ru/g extract)	Lebanon	14.3 ^b^ ± 0.1	7.5 ^c^ ± 0.0	21.2 ^a^ ± 0.5
	France	37.5 ^b^ ± 0.7	12.8 ^c^ ± 0.0	41.3 ^a^ ± 0.3
	Algeria	10.3 ^b^ ± 0.1	3.1 ^c^ ± 0.0	15.4 ^a^ ± 0.0
	Syria	30.1 ^c^ ± 0.1	36.6 ^b^ ± 0.7	39.1 ^a^ ± 0.1
TEAC (TE µmol/g extract)	Lebanon	65.4 ^b^ ± 0.1	28.2 ^c^ ± 0.1	128.3 ^a^ ± 0.7
	France	140.2 ^b^ ± 1.0	45.4 ^c^ ± 0.4	207.2 ^a^ ± 0.2
	Algeria	34.8 ^c^ ± 0.5	36.4 ^b^ ± 0.3	97.6 ^a^ ± 0.5
	Syria	127.3 ^b^ ± 0.1	124.7 ^c^ ± 0.3	165.4 ^a^ ± 0.4

^a–c^ Mean values (of three replicates) followed by different letters in the same line are significantly different.

**Table 7:** Minimum inhibitory concentration (MIC) and minimum bactericidal concentration (MBC) mean values of delipidated and hydrodistillated cumin cakes of French origin, and aromatic water.

Trait	Bacterial Strain	Delipidated Cake	Hydrodistillated Cake	Aromatic Water
MIC (mg/mL)	*Staphylococcus aureus*	0.33	>0.30	>0.17
	*Enterococcus faecalis*	0.33	>0.30	>0.17
	*Staphylococcus epidermidis*	0.08	>0.30	>0.17
	*Escherichia coli*	0.16	>0.15	>0.17
	*Pseudomonas aeruginosa*	0.33	>0.15	>0.17
MBC (mg/mL)	*Staphylococcus aureus*	0.33	>0.30	>0.17
	*Enterococcus faecalis*	0.33	>0.30	>0.17
	*Staphylococcus epidermidis*	0.08	>0.30	>0.17
	*Escherichia coli*	0.16	>0.15	>0.17
	*Pseudomonas aeruginosa*	0.33	>0.15	>0.17
